# Supplementary figures and images for: High prevalence of malaria in pregnancy among women attending antenatal care at a large referral hospital in northwestern Uganda: A cross-sectional study
Source: PLoS One. 2023 Apr 5;18(4):e0283755. doi: 10.1371/journal.pone.0283755 (PMC10075480; doi:10.1371/journal.pone.0283755)

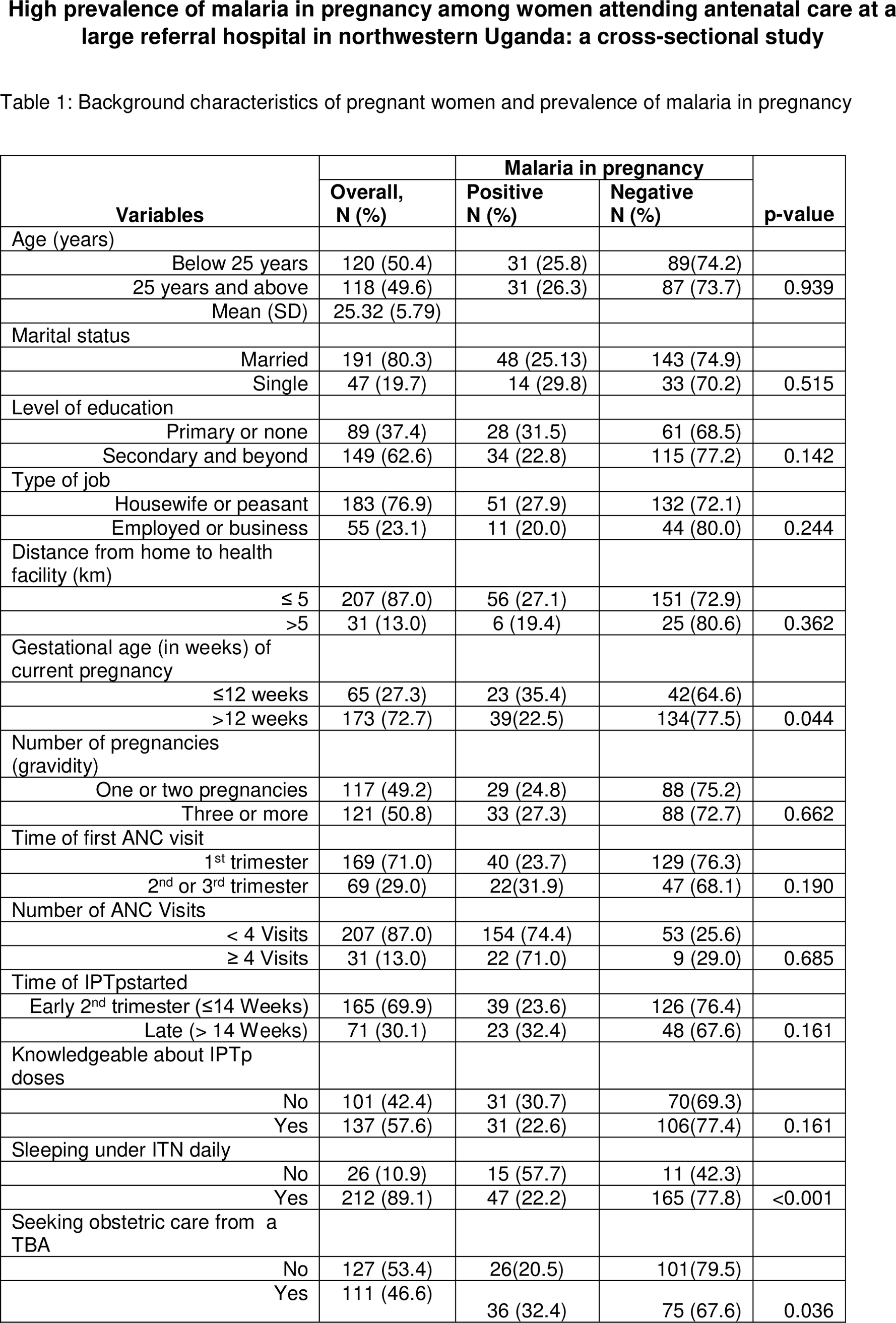

Supplement: S1 Table — (TIF) [file pone.0283755.s001.tif]

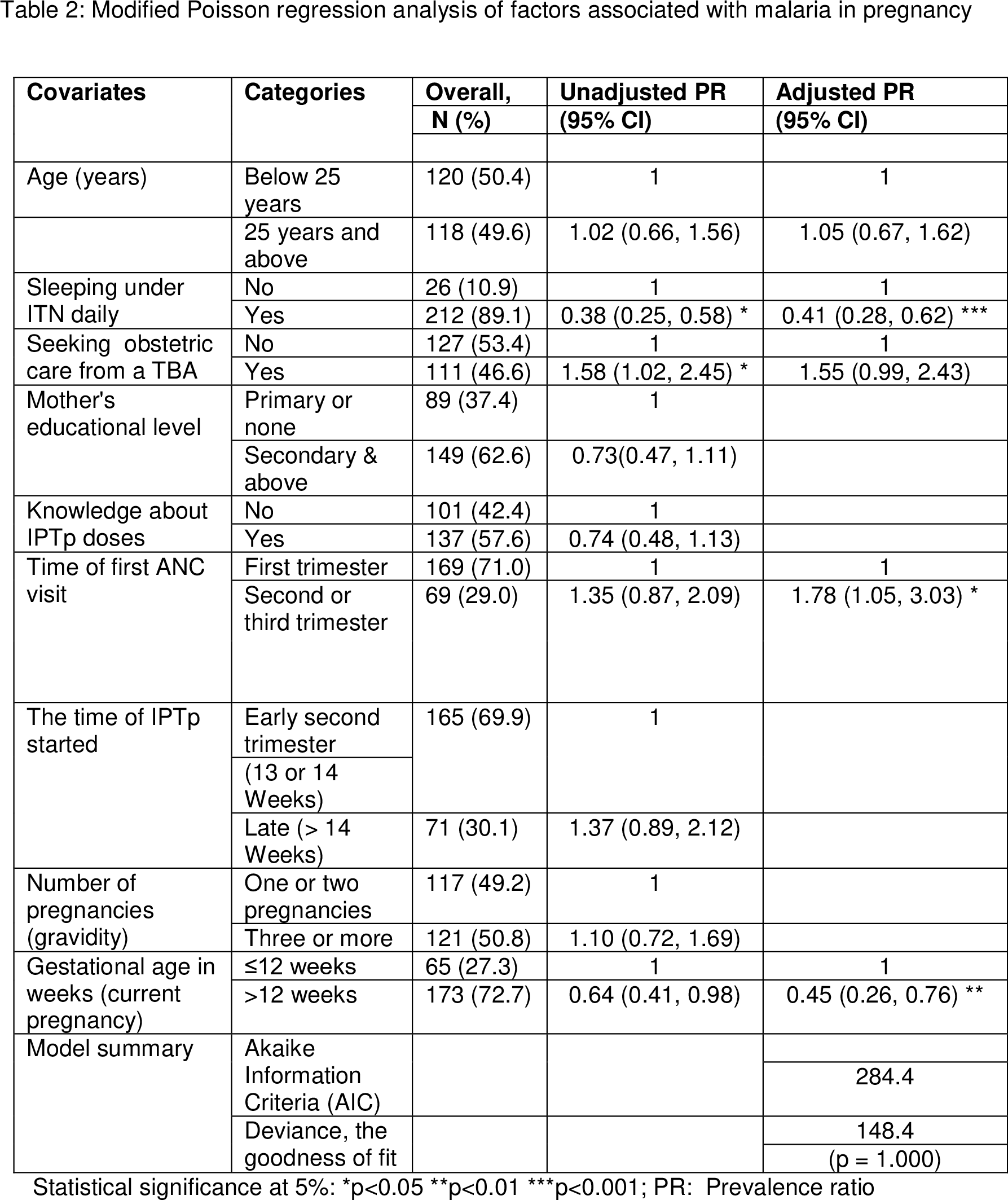

Supplement: S2 Table — (TIF) [file pone.0283755.s002.tif]

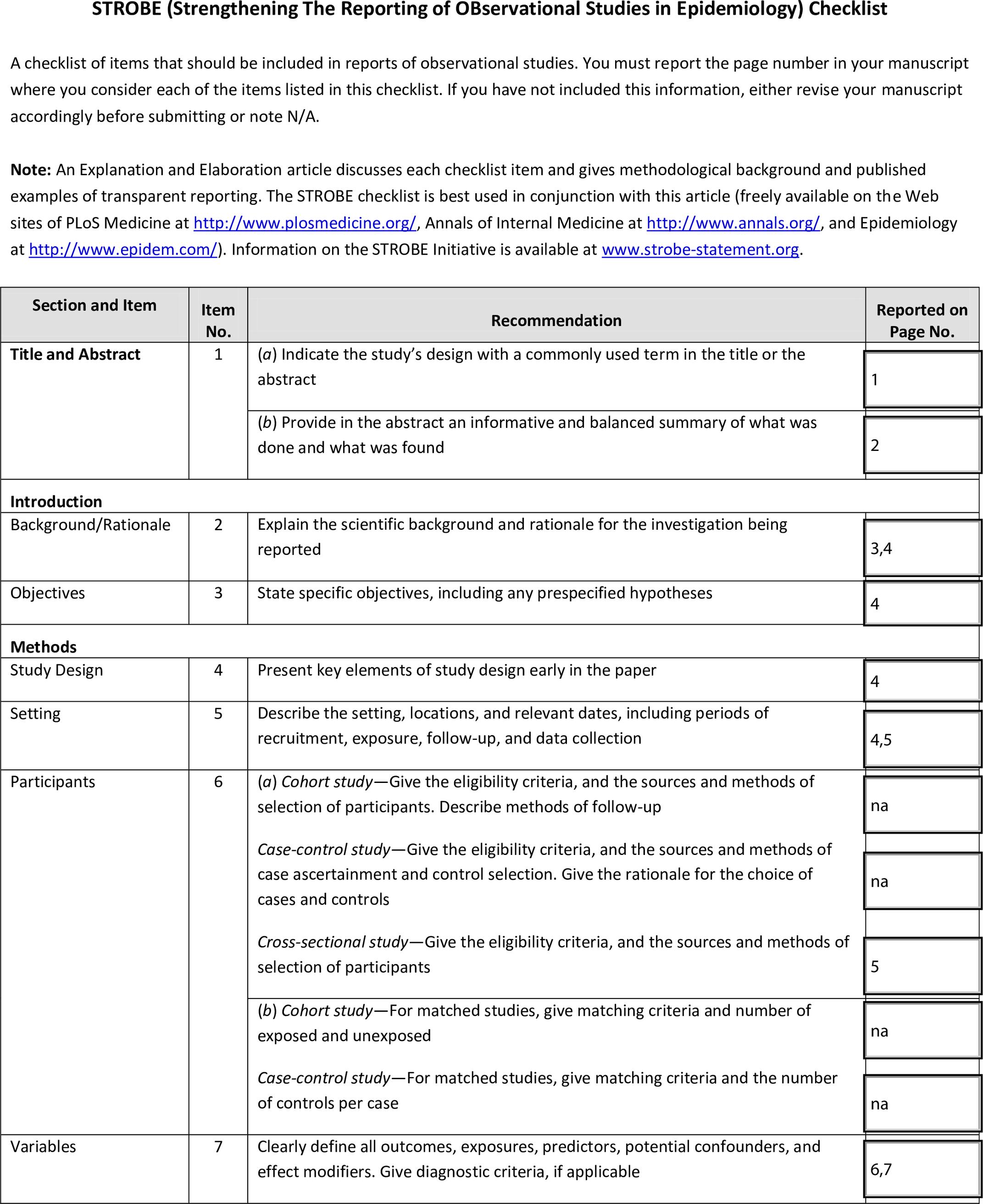

Supplement: S1 Checklist — (TIFF) [file pone.0283755.s003.tiff]
