# Supplementary material for: High prevalence of malaria in pregnancy among women attending antenatal care at a large referral hospital in northwestern Uganda: A cross-sectional study
Source: PLoS One. 2023 Apr 5;18(4):e0283755. doi: 10.1371/journal.pone.0283755 (PMC10075480; doi:10.1371/journal.pone.0283755)
Supplement: Attachment — Submitted filename: Reviewer comment_PONE-D-22-12136_19June2022.docx [file pone.0283755.s006.docx]

**Reviewer comment for PLOS ONE**

Date of review: 19 June 2022

Manuscript Number: PONE-D-22-12136

Article Type: Research Article

Full Title: High prevalence of malaria in pregnancy among women attending antenatal care at a large referral hospital in northwestern Uganda: a cross-sectional study

Corresponding Author: Ismail D Legason, MPH, Muni University, Arua, UGANDA

**Reviewer comment**

Summary of the research and overall impression

This manuscript aimed to identify the prevalence of MiP and factors associated with MiP among pregnant women in Western Uganda. Introduction should be strengthen with more literature review and presenting the burden of malaria and MiP in Uganda. Methodology and analysis is reasonable. Results session needs revision in text description and background variables analysis should be revisited. Discussion is very weak and required major revision. It needs to improve in terms of approach to discussion, referencing and citation, literature review and linked to the key findings, etc. Academic and scientific English language in presenting and discussing throughout the manuscript is also weak and required proper langue editing.

Discussion on specific area of improvement

Abstract

1. Abstract is presenting clearly in study design and methodology, data analysis methods, key findings of prevalence and factors associated with MiP. No further comment for the abstract.

Introduction

2. Burden of MiP should be emphasized in introduction. Overall prevalence of MiP and burden of malaria in Ugenda should be mentioned.
3. The patho-physiology of MiP describing the inflammatory cellular changes is not mandatory to present in the first paragraph.
4. Additional literature for the risk factors for MiP should be added. There is only one study referencing for independent risk factors for MiP.
5. Line 93: please identify the prevalence of pregnant women who present with malaria should be mentioned from the review of routine health facility data, rather than describing “the majority”.

Methods

6. Line 111 (design and setting): pls describe the general of climate setting and specify how the climate setting is described in the manuscript, eg. in figure, or in table, or in appendix. Using “elsewhere” and adding reference (23) is not appropriate.
7. Line 117 (sampling): Why do you use “combination of systematic and simple random sampling”?
8. Line 120 (sampling): “Women over 10 days” of what?
9. Sampling: It is unclear when the women were enrolled for the data collection, at the time of ANC visit OR during the following visits after sampling approach was applied OR else. Please explain.
10. Data collection: it is assumed, face-to-face interview using paper-based data collection. Please clarify.
11. Line 135 (measurement): why do you categorize the age as <25 and >=25 years.
12. Line 145 (measurement): what is the duration or timepoint defined for “sleeping under bednet” variable? eg. last night or during last week or during last month, etc?
13. Line 152: the title “statistical issue” is not relevant here. Please delete.
14. Did the measurement variables include any fever or any malaria suggested symptoms at the time of data collection, during ANC visit. If the data is available, pls include the information. Therefore, we can conclude that the cohort of MiP in the study population is symptomatic or asymptomatic. Usually, body temperature should be measured at the hospital during ANC visit; may be for the screening purpose.
15. Ethical consideration: pls mention how the written consent process was approached or applied for those pregnant women who are under-aged (<18 years).

Results

16. Line 204: the sentence should not start with the numbers (which is now starting with “173….”). Pls revise.
17. Table 1.
    a. It is presenting the “**background** characteristics of pregnant women and **prevalence** of MiP”. Pls change the title of the table and in the text.
    b. N=238 can be mentioned at the top row of the second column, not necessary to include in the first column of each variable.

Discussion

18. Line 251: using RDT is not “methodologically”, it is about the “diagnostic methods”. Please revise.
19. Line 253: what is describe about the limitation of RDT. Pls mention, not just cited to “elsewhere (33)”
20. Line 260: pls spell out “PCR”.
21. Please discuss why this study used RDT only, not combination with smear microscopy or other diagnostic methods.
22. Line 271-276: the discussion around the use of ITN regarding physical and chemical mechanism is not relevant discussion for the key finding of sleeping under bednet. On the other hand, there is no reference citation for these descriptions. Pls revise the discussion relevant for the use of ITN for this paragraph (line 268-279).
23. Line 288-289: “Relatedly, the finding that malaria in pregnancy is more likely in the first trimester is consistent with the findings of xxxxxx” is contradict with the key findings from the earlier description of the paragraph. Please check and revise as necessary.
24. Age variable should be discussed in the discussion, even though “age” is not statistically significant. It could be due to how the author categorize age (<25 and >=25). Why do you make the cut-off point with 25? Would that be any difference if the author re-categorize the age variable in a different way? It is the demographic characteristics, it should include in the final model, as well.
25. Line 299: what is meant by “modern obstetric care”?
26. Line 296-304: the discussion in this paragraph is not scientifically sound. Please revise.
27. Line 307-308: “to the best of our knowledge” and “first few studies in the region” – these descriptions are not proper academic description. And this is not the strength of the study. Pls revise.

Conclusion

28. Conclusion is weak. Pls revise.

Recommendation

I would like to propose to **MAJOR REVISION** for this manuscript.
