# Supplementary material for: High prevalence of malaria in pregnancy among women attending antenatal care at a large referral hospital in northwestern Uganda: A cross-sectional study
Source: PLoS One. 2023 Apr 5;18(4):e0283755. doi: 10.1371/journal.pone.0283755 (PMC10075480; doi:10.1371/journal.pone.0283755)
Supplement: Attachment — Submitted filename: Reviewer comment_PONE-D-22-12136_R1_12Nov2022.docx [file pone.0283755.s008.docx]

**Reviewer comment for PLOS ONE**

Date of review: 12 Nov 2022

Manuscript Number: PONE-D-22-12136_R1

Article Type: Research Article

Full Title: High prevalence of malaria in pregnancy among women attending antenatal care at a large referral hospital in northwestern Uganda: a cross-sectional study

Corresponding Author: Ismail D Legason, MPH, Muni University, Arua, UGANDA

**Reviewer comment**

Thank you for revising the manuscript, addressing all the comments and it reads the scientifically sound paper. Only a few minor comments as followed.

1. The revision for reviewer’s comments was addressed in the authors response, and can be seen in track changes version. But those changes are not accepted in the clean version, meaning the clean version is the same as the original one. It is difficult to read the track change version for the reading of entire manuscript.
2. Please make sure the scientific terminologies are used consistently. For instance, (1) “blood slide microscopy” is not the correct terminology, it should be “blood smear microscopy” throughout the manuscript. (2) malaria parasite species (P.falciparum) should be “*italic”.*
3. Table 1. Column 3,4. Please revise - MIP “positive” and “negative”, rather than “yes” and “no”.
4. Authorship: in addition to the first author, there are two senior co-authors and corresponding author is the last author. Please clarify “author’s contribution”.

Recommendation

I would like to propose to **MINOR REVISION** for this manuscript.
