# Supplementary material for: High prevalence of malaria in pregnancy among women attending antenatal care at a large referral hospital in northwestern Uganda: A cross-sectional study
Source: PLoS One. 2023 Apr 5;18(4):e0283755. doi: 10.1371/journal.pone.0283755 (PMC10075480; doi:10.1371/journal.pone.0283755)
Supplement: Attachment — Submitted filename: Response to Reviewers.docx [file pone.0283755.s009.docx]

**Response to Reviewers comments: manuscript Number: PONE-D-22-12136_R1**

**Dear Editor,**

We thank the reviewers for the constructive feedback and comments regarding our manuscript titled **“High prevalence of malaria in pregnancy among women attending antenatal care at a large referral hospital in northwestern Uganda: a cross-sectional study.”** We are pleased to submit a revised manuscript for your review and consideration.

**Review Comments to the Author**

1. Reviewer #1: Malaria in pregnant women is a relevant public health issue. It is a disease that can be prevented, treated and controlled, and therefore, it is unacceptable that there are still maternal or child deaths from malaria anywhere in the world. Therefore, this study is relevant for understanding the burden of disease that malaria in pregnant women still causes.

In the first paragraph of the results, the presentation of absolute numbers and percentages worsened in relation to the initial submission. The suggestion to change the initial submission was to present as an absolute number the research participants with the characteristic that was being presented and, in parentheses, the percentage with a standardized number of decimal places; example: 238 pregnant women were recruited, of which 62 (26.1%) were positive for malaria. The comment that requested correction in the initial submission was motivated by an error that showed the number 238 as corresponding to 76.9% and not to 100.0% of the sample.

**Author response: This error is now corrected and it reads as follows:**

**“We studied 238 women with a mean age of 25.32±5.79 years who had attended the ANC clinic; all were not malaria symptomatic cases. Of these, 207 (87.0%) resided within a 5 km radius of the health facility, 191 (80.3%) were married, 149 (62.6%) attained at least a secondary level of education, and 183 (76.9%) were unemployed. Also, 173 (72.7%) were in their second or third trimester, and 117 (49.2%) were pregnant for the first or second time. The majority of the women, 169 (71.0%), started ANC in the first trimester, 71 (30.1%) began IPTp late (> 14 weeks of pregnancy), 137 (57.6%) had adequate knowledge about the correct IPTp doses, 212 (89.1%) reported sleeping under ITNs every day, and 111 (46.6%) reported seeking obstetric care from a traditional birth attendant (TBA) during the current pregnancy. Overall, 62 (26.1%) of the participants had malaria in pregnancy by rapid diagnostic tests (RDT), with all the malaria-positive cases (62 or 100.0%) due to *P. falciparum* infection*.***

**In Table 1, our data show that malaria in pregnancy was prevalent in the first trimester compared to the second or third trimester (35.4% versus 22.5%) and among women who attended less than four ANC visits compared to those who had attended ≥4 ANC visits (74.4% versus 71.0%, respectively). However, malaria in pregnancy was less common among pregnant women aged <25 years compared to those aged ≥25 years (25.8% versus 26.3%, respectively), those in the category of one or two pregnancies versus three or more (24.8% versus 27.3%, respectively), those that had ANC visit in the first trimester (≤12 weeks) versus later trimester (>12 weeks) (23.7% versus 31.9%, respectively) and those that had early IPTp initiation (≤14 weeks) versus late IPTp initiation (>14 weeks) (23.6% versus 32.4%, respectively). Being in the first trimester (*p=0.044*), daily ITN use (*p<0.001*), and seeking obstetric care from a traditional birth attendant (*p=0.036*) showed significant differences in malaria in pregnancy in the bivariate analysis.”**

2. In the tables, while the global number is the sum of the different rows in the same column, when stratified between who has and who do not have malaria, the percentages are added in the different columns that make up the same row. This difference in the treatment of data in the same table makes it less understandable.

**Author response: Thank you for this comment. We have computed and reported row percentages instead of column percentages in the revised Table 1.**

3. Regarding the limitations of the present study, this reviewer considers that an important limitation to be discussed is the possibility that the study underestimated the size of the problem if we consider that: 1. The study was recruited during only three months of the year; 2. The study recruited research participants at a time that is not the most prevalent in malaria cases; 3. Prenatal coverage is probably very low at the study site, which allows us to infer that a high percentage of potentially infected pregnant women who do not undergo prenatal care did not have the opportunity to enter the study; 4. The study did not include symptomatic cases; 5. The study made a case diagnosis, with a low sensitivity method, so that some cases could be wrongly classified as uninfected if the parasite density was below the value necessary for the diagnosis. 6. Those who seek prenatal care are likely to be pregnant women who are most protected from malaria and other diseases.

**Author response: We have revised the study strengths and limitations sections along the suggestions and it reads:**

**“Our study has several strengths and limitations. First, we selected a random sample from a large tertiary care facility which is more likely to be heterogeneous. Hence, our results can be generalized to the population under study. Second, we controlled for interviewee response bias by verifying self-reported data against ANC records. Third, we used the malaria indicator survey toolkit, a validated tool with excellent psychometric properties, to collect all the data in the study. Our study had limitations that should be considered in the interpretation of the findings, and they include 1) A relatively small sample, which might have reduced the statistical power to detect a true association in the population; 2) The study recruitment period was relatively short (October to December or three months), and it coincided with moderate malaria transmission intensity. The malaria transmission intensity in our setting peaks around August and September every year, so the study might have underestimated the burden of malaria in pregnancy; 3) The ANC coverage in our setting was approximately 30.0%, which is lower than the recommended ≥4 ANC visits, implying that a high percentage of potentially malaria-infected pregnant women had missed the opportunity to participate in the study.**

**This might have led to an inaccurate estimate of the prevalence of malaria in pregnancy in the study; 4) The study did not include symptomatic malaria cases, and more importantly, there is the possibility that the pregnant women who sought ANC are likely those most protected from malaria and other diseases; 5) The study lacked explanatory variables, such as the reasons for not sleeping under an insecticide-treated net daily among the participants as these were not within the scope of the study; and, 6) malaria in pregnancy was diagnosed with a low-sensitivity method so some cases might have been erroneously classified as uninfected if the parasite densities were below the detection limits. Nonetheless, our results provide valuable insights and should be cautiously interpreted to guide malaria interventions among pregnant women.”**

4. Regarding the determinants, the choice of cutting off the age of analysis in pregnant women under 25 years of age and over 25 years of age concealed the possibility of analyzing adolescence (under 20 years of age) as a risk factor for infection and disease.

**Author response: We thank the reviewer for this comment. The choice of the age cut-off was informed by the distribution of data, especially the proportion of teenage pregnancies in the study. Our data show that less than 18% of the participants were below the age of 20 years. By using this cut-off, we avoided data sparsity (fewer number of people in each row or column), allowing a more meaningful statistical analysis. We request to maintain the age categories.**

Reviewer #3: Thank you for revising the manuscript, addressing all the comments and it reads the scientifically sound paper. Only a few minor comments as followed.

1. The revision for reviewer’s comments was addressed in the authors response, and can be seen in track changes version. But those changes are not accepted in the clean version, meaning the clean version is the same as the original one. It is difficult to read the track change version for the reading of entire manuscript.

**Author response: Thank you. We have ensured that the clean and tracked versions of the manuscript are now the same in the revised submission.**

2. Please make sure the scientific terminologies are used consistently. For instance, (1) “blood slide microscopy” is not the correct terminology, it should be “blood smear microscopy” throughout the manuscript. (2) malaria parasite species (P.falciparum) should be “italic”.

**Author response: We have used “blood smear microscopy” throughout the manuscript, and have deleted “blood slide microscopy”. We have italicized *“P.falciparum”* in the revised manuscript.**

3. Table 1. Column 3,4. Please revise - MIP “positive” and “negative”, rather than “yes” and “no”.

**Author response: Malaria in pregnancy has been revised as positive or negative in the tables. Please note that we have not used “MiP” anymore to easy the reading. We have used “Malaria in pregnancy” instead**.

4. Authorship: in addition to the first author, there are two senior co-authors and corresponding author is the last author. Please clarify “author’s contribution”.

**Author response: We thank the reviewer for identifying these inconsistencies. We have described the author contributions per the journal requirements. The inconsistencies have been addressed. We now have one corresponding and senior author in the revised submission.**
