# Supplementary material for: High prevalence of malaria in pregnancy among women attending antenatal care at a large referral hospital in northwestern Uganda: A cross-sectional study
Source: PLoS One. 2023 Apr 5;18(4):e0283755. doi: 10.1371/journal.pone.0283755 (PMC10075480; doi:10.1371/journal.pone.0283755)
Supplement: Attachment — Submitted filename: Response to Reviewers.docx [file pone.0283755.s010.docx]

**Response to Reviewers comments: manuscript Number: PONE-D-22-12136R2**

**Dear Editor,**

We appreciate the reviewers' insightful criticism and remarks on our paper, which is titled “High prevalence of malaria in pregnancy among women attending antenatal care at a large referral hospital in northwestern Uganda: a cross-sectional study.”

We are happy to present a revised paper for your evaluation and assessment.

**Comments to the Author**

1. Academic Editor comment 1: Expand ITN in the abstract

**Author Response: We thank the editor for noticing this error which has now been corrected in the abstract as follows**

**“Of the participants, 173 (72.7%) were in their second or third trimester, 117 (49.2%) were first or second-time pregnant women, and 212 (89.1%) reported sleeping under insecticide-treated bednets (ITNs) every day”**

2. Academic Editor comment 2: Describe the performance of RDT (sensitivity, specificity) used and how the quality of RDT was controlled.

**Author Response: We thank the editor for this insightful comment. About sensitivity and specificity of the test used, we admit this was beyond the scope of this study which has now been added to the study limitations**

**“Rapid diagnostic assays for malaria based on the histidine-rich protein (HRP-2) have sensitivity and specificity that vary from 95% to 98.2% and 59.2 to 86.3%, respectively, in investigations including general populations. However, due mostly to placental parasite sequestration, the tests are typically less sensitive when used for malaria diagnosis in pregnant women. We admit that despite the fact that it was outside the scope of our study to evaluate the performance of histidine-rich protein (HRP2)-based rapid diagnostic tests (RDTs), this may have contributed to the study's low rate of malaria in pregnant women”.**

Regarding RDT quality control, we have now addressed this under quality control measures as follows

**“The regional laboratory participates in the ongoing national malaria external quality assessment (EQA) scheme and every quarter, a batch testing of RDTs is conducted prior to their issue for laboratory use. The regional external quality assurance team samples RDT cassettes from each batch and are tested using a pair of known positive and negative blood samples confirmed by expert microscopy and PCR. Therefore, the batch used for malaria testing in the present study passed QC testing. The lab technician performed initial testing of the patients and results were reviewed by the research assistant and entered into appropriate data collection forms”.**

3. Academic Editor comment 3: Sampling technique is not clear. Explain how a combination of systematic and random sampling technique were used

**Author Response: We thank the editor for this clarification. The revised sentence reads as follows**

**“We used a combination of systematic and simple random sampling to select the participants. Upon arrival, pregnant women were registered by the midwife, and basic biodata were collected. This information was used to construct a sampling interval. Using previous ANC pattern, we estimated that we would need ten days to enrol 238 participants at an average of twenty-four per day. We divided the number of women recorded in the ANC register by the number of women required for the interview on each day to obtain a sampling interval of five and henceforth, selected every 5^th^ person recorded in the ANC register. Using the registration serial numbers, we then employed a simple random sampling method using a lottery approach to select the first and subsequent participants. This process was repeated daily until the required sample size was reached “.**

4. Reviewer #1: The authors did a great job of proofreading which greatly improved the manuscript. However, the choice that the authors made about the cutoff points of the different exposure variables may limit the comparability of the manuscript with the results of other studies in malaria-endemic locations. For example: having chosen to stratify the population between those younger than 25 and 25 or more years old, as well as having chosen to stratify between first and second pregnancies to compare with more than two pregnancies, limits comparing this with studies that show adolescence and first pregnancy as possible risks for malaria infection. Additionally, it would have been interesting to compare the positivity in the first prenatal evaluation with the positivity of the test in subsequent evaluations, since readers of the manuscript may wonder whether the high prevalence found in the first trimester of pregnancy suggests that, since malaria is such a disease prevalent in the study region, women can start the pregnancy already with parasitemia and who, being diagnosed in the first prenatal evaluation, receive treatment followed by prevention with intermittent preventive treatment, what would make the positivity in subsequent visits decrease?

**Author Response: We thank the reviewer for the insightful criticism that has now helped to improve the manuscript. We have addressed the comments on the choice of our cut off for age and gravidity under study limitations as follows**

**“Not to mention, we recognize the limitations brought on by the choice of our cut-off points for variables like age and gravidity. This limits the comparability with similar studies assessing malaria infection risks from teenage pregnancy and primigravida. Nevertheless, our findings are insightful and should be carefully interpreted to direct malaria interventions among pregnant mothers”.**

The reviewer’s comment on whether women could become pregnant when they already have malaria was very insightful and we have now used it to expand our explanation for the high malaria burden in first trimester as below

**“Relatedly, the finding that malaria in pregnancy is less likely in the second or third trimester is consistent with the findings of Simon-Oke et al. in Nigeria, Accrombessi et al. in Benin, and Gontie et al. in Ethiopia. The biologically plausible reason is the lack of protection against malaria during the first few months of pregnancy due to systematic cytokine bias, which leads to weakened immunity. Yet, it is also plausible that women may contract malaria before becoming pregnant, which would explain the increased prevalence seen in the first trimester. It would be interesting to compare malaria positivity at the first prenatal appointment with the positivity of the test during subsequent visits, even if our study did not analyse data from two time points to establish this connection”.**

**We also took this opportunity to tidy up the manuscript e.g., the acknowledgement statement now reads like this**

“We thank the pregnant women who participated in the study. We are grateful to the hospital staff, particularly the nurses and midwives working in the ANC clinics who helped with data collection and availed the ANC registers, and made it possible to interview their clients. We also want to express our gratitude to the hospital management for permission to conduct the study”.
