# Supplementary material for: High prevalence of malaria in pregnancy among women attending antenatal care at a large referral hospital in northwestern Uganda: A cross-sectional study
Source: PLoS One. 2023 Apr 5;18(4):e0283755. doi: 10.1371/journal.pone.0283755 (PMC10075480; doi:10.1371/journal.pone.0283755)
Supplement: Attachment — Submitted filename: Response to Reviewers.docx [file pone.0283755.s011.docx]

**Response to Reviewers comments: manuscript Number: PONE-D-22-12136R3**

**Dear Editor,**

We appreciate your critical review of our manuscript titled “High prevalence of malaria in pregnancy among women attending antenatal care at a large referral hospital in northwestern Uganda: a cross-sectional study.”

We are pleased to share a revised manuscript with you for review.

**Comments to the Author**

Academic editor: Abstract: In this study, RDT was used to diagnose malaria not blood smear microscopy, therefore remove “blood smear microscopy” from the abstract (line #42).

**Author response: We thank the editor for the critical eye into the paper. We have now removed “blood smear microscopy” from the abstract and the new sentence reads like this**

**“The prevalence of malaria in pregnancy was 26.1% (62/238) by rapid diagnostic testing (RDT)”**

Academic editor: Materials and Methods: in subsection ‘data collection and measurement’ line #179 next to “…. by technician with >10 year experience…”add how RDT was performed in brief  and the performance of the RDT used (sensitivity, specificity,  positivity and negative predictive value)

**Author response: We again thank the editor for identifying this omission. We have addressed it and the new paragraph reads like this**

**“Briefly, about 5µL of capillary or venous EDTA blood was introduced into the sample well using the sample transfer device that came with the RDT kit. This was followed by two drops of the assay buffer to the assay buffer well. The timer was set for 20 minutes before a test result could be read. The test results were interpreted anytime from 20 but before 30 minutes to minimize inaccurate test results that occur due to reaction product deterioration. A positive result was considered if two lines appeared on the cassette, otherwise it was negative if one control line appeared. In case a test line appeared without a control line or nothing at all appeared, the test was regarded invalid and a repeat test performed on a new device. Standard laboratory practices including aseptic procedures for obtaining samples and infection control measures were observed at all times by the laboratory personnel testing the samples. The performance of the test kits, SD Bioline Malaria Ag Pf (SD Bioline Inc. Korea) were as follows; sensitivity 98.2%, and specificity 91.6%, with positive and negative predictive values of 81.8% and 99.2% respectively”**
